# The N-Terminal Region of the Ryanodine Receptor Affects Channel Activation

**DOI:** 10.3389/fphys.2017.00443

**Published:** 2017-06-30

**Authors:** Andrea Faltinova, Nataša Tomaskova, Marián Antalik, Jozef Sevcik, Alexandra Zahradnikova

**Affiliations:** ^1^Department of Muscle Cell Research, Institute of Molecular Physiology and Genetics of the Centre of Biosciences, Slovak Academy of SciencesBratislava, Slovakia; ^2^Department of Biochemistry and Structural Biology, Institute of Molecular Biology, Slovak Academy of SciencesBratislava, Slovakia; ^3^Faculty of Science, Institute of Chemical Sciences, Pavol Jozef Šafárik UniversityKošice, Slovakia

**Keywords:** ryanodine receptor, domain peptide, allosteric activation, CPVT mutations, planar lipid bilayer, structural modeling

## Abstract

Mutations in the cardiac ryanodine receptor (RyR2), the ion channel responsible for release of calcium ions from intracellular stores into cytoplasm, are the cause of several inherited cardiac arrhythmias. At the molecular level, disease symptoms can be mimicked by domain peptides from mutation-prone regions of RyR2 that bind to RyR2 and activate it. Here we show that the domain peptide DP_cpvtN2_, corresponding to the central helix of the N-terminal region of RyR2, activates the RyR2 channel. Structural modeling of interaction between DP_cpvtN2_ and the N-terminal region of RyR2 in the closed and open conformation provided three plausible structures of the complex. Only one of them could explain the dependence of RyR2 activity on concentration of DP_cpvtN2_. The structure of the complex was at odds with the previously proposed “domain switch” mechanism of competition between domain peptides and ryanodine receptor domains. Likewise, in structural models of the N-terminal region, the conformational changes induced by DP_cpvtN2_ binding were different from those induced by mutation of central helix amino acids. The activating effect of DP_cpvtN2_ binding and of mutations in the central helix could be explained by their similar effect on the transition energy between the closed and open conformation of RyR2.

## Introduction

Cardiac ryanodine receptor channels (RyR2) of the sarcoplasmic reticulum (SR) membrane open during the systole to release calcium ions for contraction, while they stay virtually closed during the diastole (Meissner, 2004). RyR2 open probability is allosterically regulated by ligand binding (Zahradnik et al., 2005; Zahradnikova et al., 2010; Tencerova et al., 2012; Petrovic et al., 2015). Multiple, distinct regions of RyR2—N-terminal (aa 1–600), central (aa 2,000–2,500) and C-terminal (aa 3,700–4,200 and 4,500–5,000)—are subject to mutations in patients with several types of hereditary arrhythmias (Yano et al., 2006; Durham et al., 2007; George et al., 2007), which are characteristic by increased diastolic calcium leak from the SR (Yano et al., 2006; Durham et al., 2007).

The large cytoplasmic part of the RyR comprises the N-terminal ~90% of the total RyR mass (Ma et al., 2004) and contains regulatory sites that modulate channel activity by binding endogenous modulators (Ca^2+^, Mg^2+^, ATP) as well as exogenous ligands (Meissner, 2004). The small C-terminal hydrophobic part contains the channel pore (Bhat et al., 1997; Ma et al., 2004). Activation of RyR is accompanied by conformational changes of its tertiary structure at several regions (Ikemoto and Yamamoto, 2002). Large-scale conformational RyR rearrangements between open and closed states have also been inferred from cryoelectron microscopy (cryo-EM) data (Orlova et al., 1996; Serysheva et al., 1999; Samso et al., 2009; des Georges et al., 2016; Peng et al., 2016; Wei et al., 2016).

Cryo-EM studies have shown that the cytoplasmic part of the RyR is composed of multiple domains (Serysheva et al., 2008). Regulation of calcium release at the molecular level is supposed to be based on conformational changes of and interaction between individual domains (El-Hayek et al., 1995; Ikemoto and Yamamoto, 2000; George et al., 2004; Yuchi and Van Petegem, 2011). The link between conformational changes and RyR2 activity is at present not sufficiently understood. Mutations at different positions of the N-terminal and central region share the mode of their action, *viz*., hyper-activation of the channel and increased sensitivity of the RyR channel to agonists (Yamamoto et al., 2000). At the same time, RyR open probability can be increased by binding of short peptides (“domain peptides,” El-Hayek et al., 1999) with a sequence identical to the wild-type sequence of a mutation-susceptible part of the N-terminal or central domain (Ikemoto and Yamamoto, 2002). In this way, the effect of several peptides from the skeletal RyR1 isoform (DP1, DP3, DP4) (Yamamoto et al., 2000) as well as from RyR2 (DP1c, DP_cpvtC_) (El-Hayek et al., 1999; Yamamoto et al., 2000; Laver et al., 2008; Tateishi et al., 2009; Faltinova and Zahradnikova, 2013) has been shown. The similarity of the effect of mutations from the N-terminal and central region on one hand, and of the effect of the respective peptides on the other hand led to formulation of a hypothesis that arrhythmogenic mutations weaken the interactions between RyR domains (the “domain zipper”), and that the domain peptide and the corresponding channel sequence compete for the binding site at the “domain zipper” (Ikemoto and Yamamoto, 2000; Yamamoto et al., 2000; Tateishi et al., 2009; Suetomi et al., 2011).

Here we have probed the role of the N-terminal region in RyR2 activation using a domain peptide from the second cluster of the N-terminal region CPVT mutations (aa 164–507), which we named DP_cpvtN2_ (hRyR2^410−438^). This peptide corresponds to the sequence of the central helix of the N-terminal region (Borko et al., 2014), which is fully conserved in RyR2 channels of several mammalian species: mouse, rat, and human (Uniprot entries E9Q401, B0LPN4, and Q92736, respectively) as well as swine (Peng et al., 2016). The central helix is located between the Pfam domains MIR and RIH, which comprise the sequences corresponding to the domain peptide DP3 and DP1, respectively (Yamamoto et al., 2000). Both DP3 and DP1 have been previously shown to affect the activity of ryanodine receptors: DP1 activates both the skeletal and the cardiac RyR isoform (El-Hayek et al., 1999), and DP3 inhibits activation of RyR1 by the central domain peptide DP4 (Yamamoto et al., 2000); their effect has been interpreted in terms of the “domain zipper” hypothesis (Ikemoto and Yamamoto, 2000, 2002; Yamamoto et al., 2000). The central helix comprising amino acids 410–438 of the human RyR2 is important for the stability of the N-terminal region (NTR) of RyR2 (Borko et al., 2014). It contains five amino acids that may undergo a total of 7 mutations causing arrhythmias (George et al., 2007), which have been proposed to diminish the stability of NTR (Borko et al., 2014). Therefore, it seems plausible that binding of DP_cpvtN2_ would weaken the stability of RyR2 and thus promote channel opening.

In single channel activity records, DP_cpvtN2_ increased RyR2 open probability by inducing long openings. Moreover, molecular models of complexes of wild type NTR, NTR central helix mutants and complexes of NTR with DP_cpvtN2_ in the open and closed conformation indicated allosteric mechanism of DP_cpvtN2_—RyR2 interaction. The allosteric interaction was distinct from the “domain-zipper” mechanism proposed by Ikemoto and Yamamoto (2002). Comparison of structural models of the peptide—NTR complex with models of central helix mutants of the NTR suggested that the similar effect of mutations and peptide-NTR interaction on RyR2 open probability is caused by decreased energy of the open/closed conformation change in the NTR.

## Materials and methods

### Preparation of SR membrane vesicles

All experimental protocols were approved by the State veterinary and food administration of the Slovak Republic (Ro-3820/05-221, Ro-2821/09-221) and by the ethical committee of the Centre of Biosciences. All methods were carried out in accordance with the European directive 2010/63/EU. Male rats (Wistar Han, 240–310 g, breeding station Dobra Voda, reg. No. SK CH 24011, Slovak Republic) were anesthetized with sodium pentobarbital (50 mg kg^−1^ i.p.), the hearts were excised, and cardiac SR microsomes were isolated from rat heart ventricles by differential centrifugation without further purification steps, as previously described (Gaburjakova and Gaburjakova, 2006; Faltinova and Zahradnikova, 2013), snap frozen in liquid nitrogen and stored at −70°C.

### Chemicals

1,2-dioleoyl-sn-glycero-3-phospho-L-serine (DOPS), 1,2-dioleoyl-sn-glycero-3-phosphatidylcholine (cis)—DOPC, 1,2-diphytanoyl-sn-glycero-3-phosphatidylcholine (DPPC), and 1,2-dioleoyl-sn-glycero-3-phosphoethanolamine (DOPE) were from Avanti Polar Lipids (USA).

The peptide DP_cpvtN2_, containing amino acids 410–438 of human RyR2 with the sequence HEESRTARVIRSTVFLFBRFIRGLDALSK was synthesized by GenScript (USA). Aliquots of a stock solution of the lyophilized peptide in water (100 μmol/l) were kept at −20°C.

### Single RyR2 channel measurements

Preparation of bilayers, incorporation of channels and electrophysiological measurements were performed as previously described (Faltinova and Zahradnikova, 2013). In brief, bilayer lipid membranes were formed over a 20–50 μm aperture in a polystyrene chamber. The aperture was first treated with a solution (26 mg/ml) of DOPS, DOPC and DPPC (3:5:5) in N-decane (Aldrich, USA). After evaporation of N-decane, the *cis* and *trans* compartments of the bilayer chamber were filled with the solutions (in mmol/l): cis, 250 HEPES, 50 KCl, 1 EGTA, 125 TRIS, 0.5 CaCl_2_, pH 7.35, [Ca^2+^] = 90 nM; trans, 30 HEPES, 50 KCl, 1 Ca(OH)_2_, 7 Ba(OH)_2_, pH 7.35, and the bilayer was formed using a mixture of DOPS and DOPE (3:10; 26 mg/ml in N-decane). After formation of a bilayer, RyR2 channels were incorporated from a suspension of microsomal vesicles of the SR, added to the cis compartment. RyR identity was checked at the beginning of the experiment at high Ca^2+^, when it was characteristic by its single-channel amplitude and open duration (Tencerova et al., 2012; Faltinova and Zahradnikova, 2013). Under the above ionic conditions, no other channels have similar properties. Detection of RyR channel was followed by perfusion to a basal Ca^2+^ level. The identity of RyR was occasionally further tested at the end of the experiment by addition of 100 μM Ca^2+^.

Currents through the incorporated single RyR2 channels were measured in voltage-clamp mode at 0 mV with MultiClamp 700B amplifier (Molecular Devices, USA) with a 50 GΩ feed-back resistor. The signal was filtered by a 4-pole Bessel filter at 1 kHz and digitized using an A/D converter (Digidata 1440A, Molecular Devices, USA) at a frequency of 4 kHz. Acquisition and analysis were performed using pClamp software (Ver. 10, Molecular Devices, USA) and Origin Pro (Ver. 9, OriginLab Corporation, USA).

### Channel gating models

Equilibrium open probabilities for different gating models (Equations 1–3) were determined from the gating schemes. Equations were derived using Mathematica (Ver. 10, Wolfram Research, USA) as previously described (Zahradnik et al., 2005). The value of EC50 was calculated in Mathematica from Equations (1) to (3) as the ligand concentration, at which the increase of open probability reached 50% of maximum increase.

### Bioinformatics

Homology models of peptides were made using the *I_TASSER* server (Roy et al., 2010) (http://zhanglab.ccmb.med.umich.edu/I-TASSER/). Modeling of loops was performed in *Modeller* (ver. 9.15) (Sali and Blundell, 1993).

Docking of protein structures into electron density maps was performed using the program *COLORES* from the *SITUS 2.7* package (Wriggers, 2010) as described previously (Borko et al., 2014).

Peptide-protein complex formation was modeled using the *GRAMM-X* public web server (Tovchigrechko and Vakser, 2006). All model structures were further energy-optimized using the *Chimera* interface of the *Molecular Modelling Toolkit* (Hinsen, 2000) with the optimization criteria as follows: 200 iterations for steepest descent gradient method followed by maximum 500 iterations for conjugated gradients method, maximum total energy difference at the last iteration of 0.015 kJ/mol per residue. Interactions between amino acids were quantified using the PIC web server (Tina et al., 2007). Hydrogen bonds were analyzed in *Chimera* with constraints according to Mills and Dean (1996) relaxed by 0.4 Å and 30°.

### Data analysis

Statistical analysis and data fitting were performed in Origin Pro (Ver. 9, OriginLab Corporation, USA). Analysis of linear models was performed in Mathematica (Ver. 10, Wolfram Research, USA).

### CD spectroscopy

Circular dichroism (CD) measurements were performed using a Jasco J-810 spectropolarimeter (Japan). CD measurements were performed in the 198–250 nm wavelength range, which enabled estimation of the secondary structure of the peptide. The DP_cpvtN2_ peptide was dissolved either in 2 mM Na-phosphate (pH 7.0) or in 99.8% ethanol to a final concentration of 1.5–7 μM. CD spectra were expressed as averages of 3–5 consecutive scans. 1 mm cuvette was used for measurements. Typically, a time constant of 2 s, bandwidth of 2.0 nm, scanning speed 200 nm/min and the standard sensitivity were used. The spectra were analyzed using the web server CAPITO (Wiedemann et al., 2013), which provides the percentage of α-helical, β-sheet and random coil in the secondary structure.

## Results

### Activation of RyR2 by DP_cpvtN2_ under diastolic conditions

**Figure 1:**
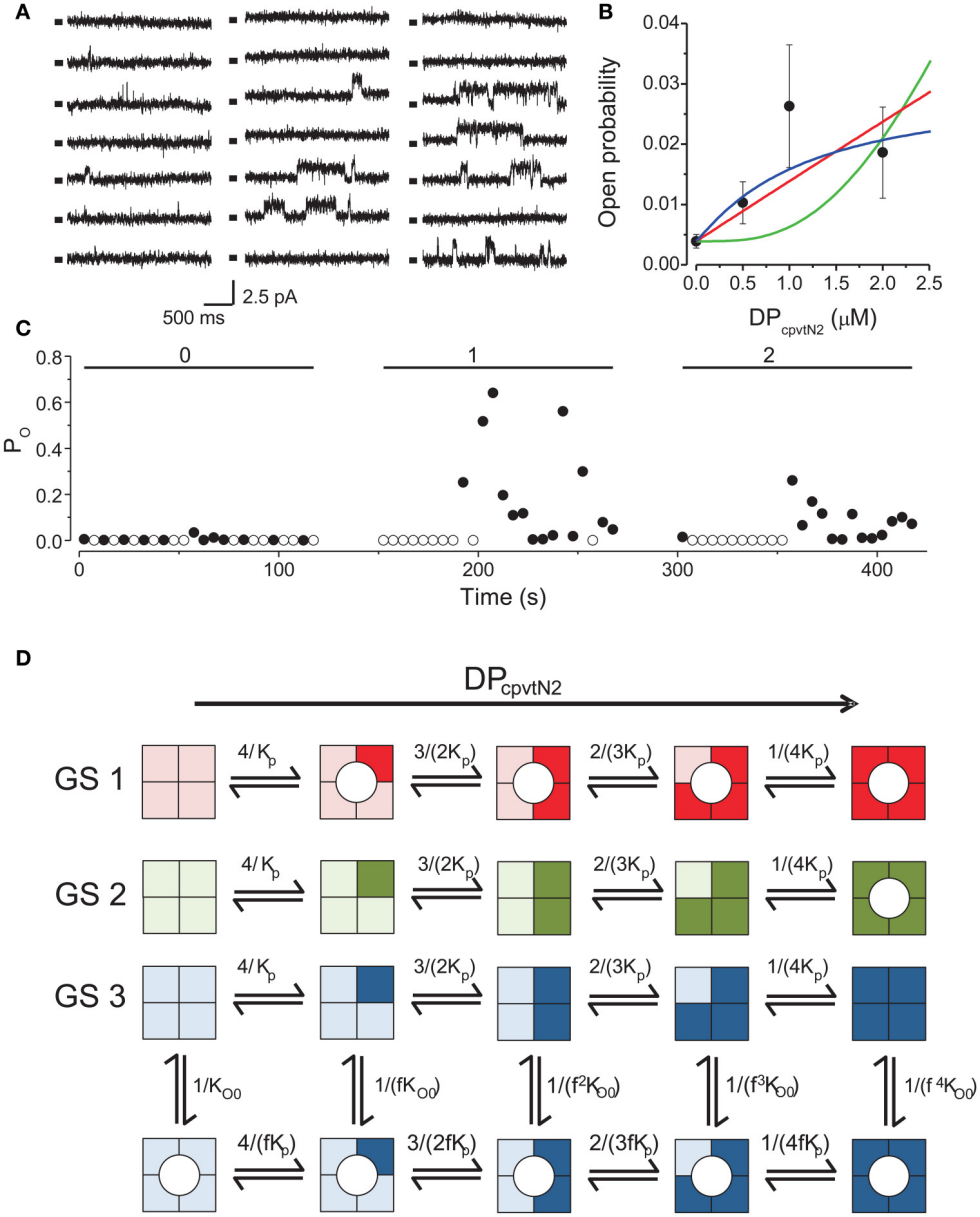
Activation of RyR2 by the peptide DP_cpvtN2_. **(A)** RyR2 activity at 0 mV under control conditions (left) and after addition of 1.0 μM (center) or 2.0 μM DP_cpvtN2_ (right). All records correspond to the same single channel. Markers indicate the closed state of the channel. **(B)** The concentration dependence of the effect of the domain peptide DP_cpvtN2_ on the open probability of the RyR2 channel (*n* = 9 channels). Lines illustrate the fit of the data by gating schemes GS 1A (red; Equation 1), GS 1B (green; Equation 2) and GS 2 (blue; Equation 3) with parameters from Table 1. **(C)** Time course of open probability changes induced by DP_cpvtN2_ in the RyR channel shown in **(A)**. The average open probability measured in 5-s segments is plotted against time after channel incorporation. Concentration of DP_cpvtN2_ (in μM) is shown at the top. **(D)** Possible models of activation of the RyR2 by the peptide. Monomers with bound peptide are shown in a darker shade. Peptide-induced open state is denoted by a circle. GS 1 and GS 2 assume that the binding of peptide induces channel opening directly if any of the monomers is peptide-bound (GS 1) or if all four monomers have to be occupied by the peptide to induce channel opening (GS 2). GS 3 assumes an allosteric effect of the peptide, i.e., increase of the relative stability of the open state with increasing number of peptide-bound monomers. The formulae at individual reaction steps indicate the respective stepwise stability constants. The integers in the formulae in the numerator and denominator reflect the number of monomers undergoing the forward and backward transition, respectively. The names of the variables are explained in the legend to Equations (1)–(3).

The activity of single RyR2 channels was recorded at cytosolic (100 nM) and luminal calcium (1 mM) concentrations and at a holding potential (0 mV) corresponding to the diastolic conditions. Under these conditions, the open probability of the ryanodine receptor was very low (P_O_ = 0.0039 ± 0.0012, *n* = 9) and consisted of short openings (t_O_ ~ 15 ms). Aliquots of DP_cpvtN2_ stock solution were sequentially added into the *cis* compartment up to final concentrations in the range of 0.5–2 μM. The activity of the channel under control conditions and after each increase of DP_cpvtN2_ concentration was monitored for a 2-min. period. Typical current traces are shown in Figure 1A. Within 1 min after addition of DP_cpvtN2_, sporadic occurrence of long openings (t_O_ >100 ms) could be observed. Open probabilities at different concentrations of DP_cpvtN2_ obtained from 9 channels are summarized in Figure 1B. Increase in open probability and the presence of long openings could be observed at all DP_cpvtN2_ concentrations in the range of 0.5–2 μM; long openings (t_O_ > 100 ms) were never observed in the absence of DP_cpvtN2_. There was a high temporal variation of channel open probability (Figure 1C) as well as of the responses of individual channels (Figure 1B). Therefore, it was not directly apparent whether a maximal effect of DP_cpvtN2_ was achieved at the highest DP_cpvtN2_ concentration of 2 μM. Higher DP_cpvtN2_ concentrations could not be used, because they induced rupture of the bilayer membrane after DP_cpvtN2_ addition. The median time to rupture at 1–2 μM DP_cpvtN2_ did not depend on the presence of RyR in the membrane, and in the absence of RyR2 the rupture was not preceded by channel-like activity.

We compared the observed calcium dependence of open probability with the predictions of three putative gating schemes (GS1–GS3) describing channel activation, illustrated in Figure 1D, in which DP_cpvtN2_ sequentially binds to individual monomers of the RyR2 tetramer. According to GS1, binding of one DP_cpvtN2_molecule to the ryanodine receptor is sufficient for channel opening, and the long channel openings correspond to the periods of bound DP_cpvtN2_. The concentration dependence of open probability (P_O_) is then described by the equation:

(1) $$\begin{array}{l} {\text{P}}_{\text{O}}={\text{P}}_{\text{O}0}+(1-{\text{P}}_{\text{O}0}){\text{c}}_{\text{p}}({\text{c}}_{\text{p}}+2{\text{K}}_{\text{p}})({\text{c}}_{\text{p}}^{2}+2{\text{c}}_{\text{p}}{\text{K}}_{\text{p}}+2{\text{K}}_{\text{p}}^{2})\\ /{\left({\text{c}}_{\text{p}}+{\text{K}}_{\text{p}}\right)}^{4}, \end{array}$$

where P_*O*0_ is the open probability in the absence of DP_cpvtN2_, c_p_ is the concentration of DP_cpvtN2_ and K_p_ is its dissociation constant.

According to GS2, the channel opens only upon binding of DP_cpvtN2_ to all four RyR2 binding sites. The concentration dependence of open probability (P_O_) is then described by the equation:

(2) $${\text{P}}_{\text{O}}={\text{P}}_{\text{O}0}+(1-{\text{P}}_{\text{O}0})\;{\text{c}}_{\text{p}}^{4}/{\left({\text{c}}_{\text{p}}+{\text{K}}_{\text{p}}\right)}^{4},$$

where individual parameters were explained previously.

According to GS3, DP_cpvtN2_ increases RyR2 open probability allosterically, as previously observed for the peptide DP_cpvtC_ (Faltinova and Zahradnikova, 2013). That is, when DP_cpvtN2_ is bound to the channel, the free energy necessary for channel opening is decreased. In this scheme, the concentration dependence of open probability (P_O_) is described by the equation:

(3) $${\text{P}}_{\text{O}}={\left({\text{c}}_{\text{p}}+{\text{f}}_{\text{p}}{\text{K}}_{\text{p}}\right)}^{4}/({\left({\text{c}}_{\text{p}}+{\text{f}}_{\text{p}}{\text{K}}_{\text{p}}\right)}^{4}+{\text{f}}_{\text{p}}^{4}{\left({\text{c}}_{\text{p}}+{\text{K}}_{\text{p}}\right)}^{4}(1/{\text{P}}_{\text{O}0}-1)),$$

where f_p_ is the allosteric factor that couples DP_cpvtN2_ binding to channel opening, and the remaining parameters were described previously.

The best data fit curves corresponding to Equations (1)–(3) are shown in Figure 1B, and the fit parameters and EC_50_ values are shown in Table 1.

**Table 1:** Parameters and variables of gating models characterizing interaction between DP_cpvtN2_ and RyR2.

	**GS1**	**GS2**	**GS3**
**PARAMETER**			
P_O0_	0.0039	0.0039	0.0039
K_p_ (μM)	351 ± 99	3.35 ± 0.56	0.30 ± 0.54
f_p_	N/A	N/A	0.61 ± 0.10
**VARIABLE**			
P_Omax_	1	1	0.0275
χ^2^	3.3 × 10^−4^	4.0 × 10^−4^	3.1 × 10^−4^
DF	29	29	28
EC50(μM)	66.4	17.6	0.41

*P_O0_, Open probability in the absence of DP_cpvtN2_; K_p_, dissociation constant of DP_cpvtN2_; f_p_, allosteric factor; P_Omax_, maximum open probability; DF, number of degrees of freedom; EC50, 50% effective concentration*.

Visually, the fit of the data by GS1 is acceptable while that by GS2 shows systematic deviation. This was confirmed by analysis of the likelihood of the fit which rejected GS2 (Akaike weight 0.92 and 0.08 for GS1 and GS2). GS1 and GS3 provided acceptable fits and both are in accordance with the effect of DP_cpvt2_ on RyR2 open probability (Akaike weight 0.52 and 0.48 for GS1 and GS3, respectively).

However, the data in Table 1 suggest that GS1 and GS3 lead to diametrically opposed mechanism of peptide action: according to GS1, DP_cpvtN2_ has very low affinity to the RyR2 monomer that is independent of the number of DP_cpvtN2_ molecules bound to the RyR2 tetramer. That is, the free energy of DP_cpvtN2_ binding is expected to be positive even when the channel resides in the peptide-induced open state. At the same time, DP_cpvtN2_ has a very strong effect on open probability, i.e., the free energy of channel opening is expected to be much lower in the complex than in the free monomer.

On the contrary, according to GS3 (Table 1, Figure 1D), the DP_cpvtN2_ has high affinity to RyR2, i.e., the free energy of DP_cpvtN2_ binding is expected to be negative. The effect on RyR2 open probability is modest, thus the free energy of channel opening is expected to be somewhat lower for the complex than for the free RyR2 monomer. Affinity of DP_cpvtN2_ is higher in the open than in the closed states by a factor of 1/f_p_, and thus the free energy of complex formation is expected to be lower in the open than in the closed state.

### Model of DP_cpvtN2_ binding to the ryanodine receptor

Mutations in the central helix decrease the thermal and conformational stability of NTR and/or destabilize the interaction between domains A, B, and C of the N-terminal region (Kimlicka et al., 2013b; Borko et al., 2014). Furthermore, mutation R402G of the skeletal RyR1 isoform, corresponding to R417 in the central helix of NTR in hRyR2, induces changes in the relative orientation of domains A and C of the N-terminal region (Kimlicka et al., 2013a). The “domain switch” mechanism (Ikemoto and Yamamoto, 2000; Yamamoto et al., 2000; Tateishi et al., 2009; Suetomi et al., 2011) assumes that DP_cpvtN2_ exhibits its effect by competition between DP_cpvtN2_ and central helix for a common binding site that might involve domains A/B. This mechanism would require three conditions to be met:

1. The conformation of DP_cpvtN2_ in solution should be similar to the conformation of the central helix.
2. Formation of a complex between DP_cpvtN2_ and the channel molecule should be energetically plausible.
3. Upon complex formation, some contacts between the central helix and the A and/or B domains should be disrupted and analogous contacts between DP_cpvtN2_ and the A and/or B domains should appear.

**Figure 2:**
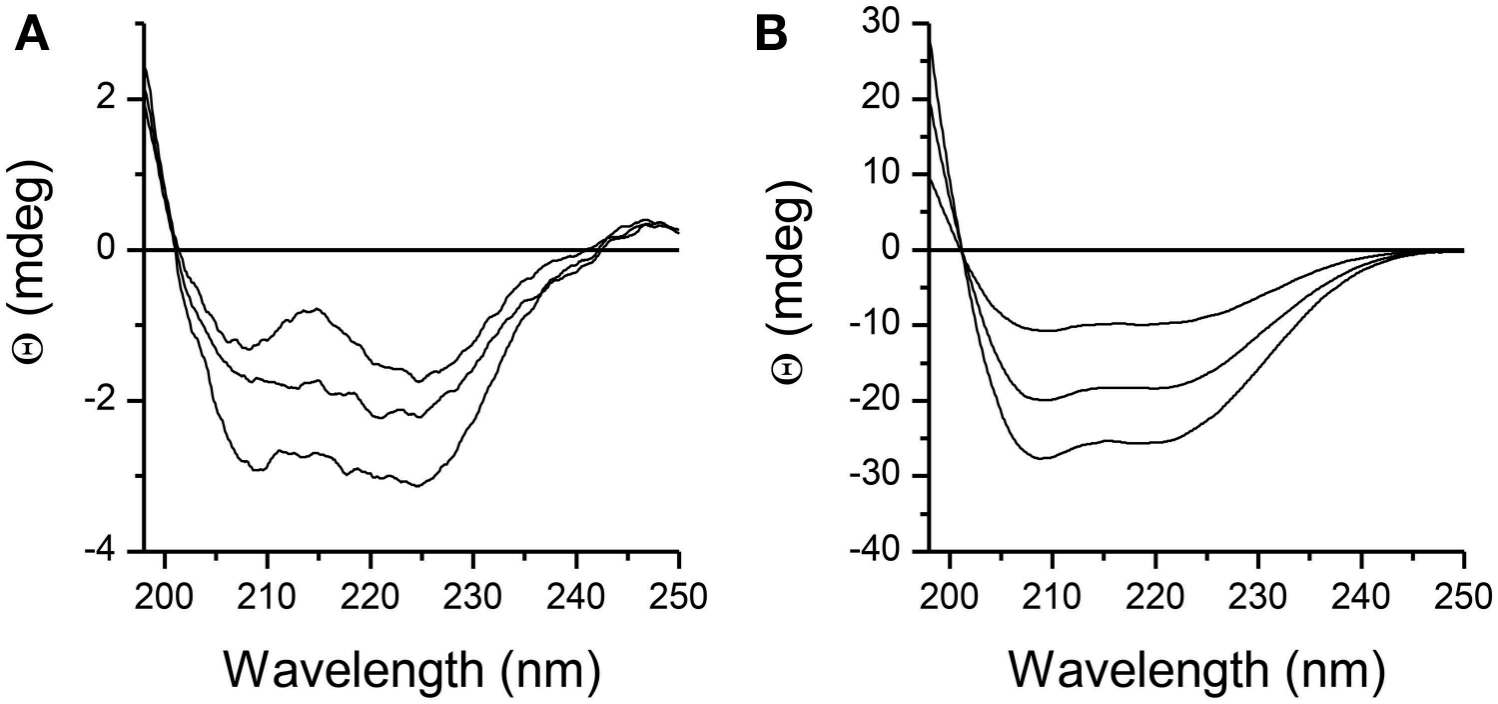
CD spectra of the peptide DP_cpvtN2_. **(A)** 1.5, 2.0, and 3.0 μM solution of DP_cpvtN2_ in phosphate buffer, pH 7.0. **(B)** 1.5, 2.0, and 3.0 μM solution of DP_cpvtN2_ in ethanol.

Structure of DP_cpvtN2_ in solution was examined by CD spectroscopy. The CD spectra of 1.5–3 μM DP_cpvtN2_ solution in water and in ethanol are shown in Figures 2A,B, respectively. The CD spectra of DP_cpvtN2_ show a positive band with a maximum below the measuring range (<198 nm) and two negative bands with minima at 208 and 225 nm that suggest that a substantial fraction of the peptide is in the α-helix conformation. In ethanol (Figure 2B), the ellipticity was an order of magnitude higher, without a substantial change in the shape of the spectra. The content of α-helix was estimated using the web server CAPITO (http://capito.nmr.leibniz-fli.de/index.php) to be 99%. The high content of α-helix estimated from CD spectra shows that the structure of DP_cpvtN2_ in solution is comparable to the structure of the central helix in the RyR2 molecule.

The interaction of DP_cpvtN2_ with the N-terminal region was studied in structural models of the closed and open conformation of the NTR (Borko et al., 2014) using the *GRAMM-X* web server. The model of DP_cpvtN2_ was constructed using the *I_TASSER* server. The model of NTR in the closed conformation was taken from Borko et al. (2014), and the model of NTR in the open conformation was constructed by aligning the A, B, C domains of the NTR model to the respective domains of 4JKQ in the open conformation (Borko et al., 2014), reconstructing the loops between domains in *Modeller*, and minimizing the energy of the resulting model in *Chimera* using the built-in *Molecular Modelling Toolkit*.

**Figure 3:**
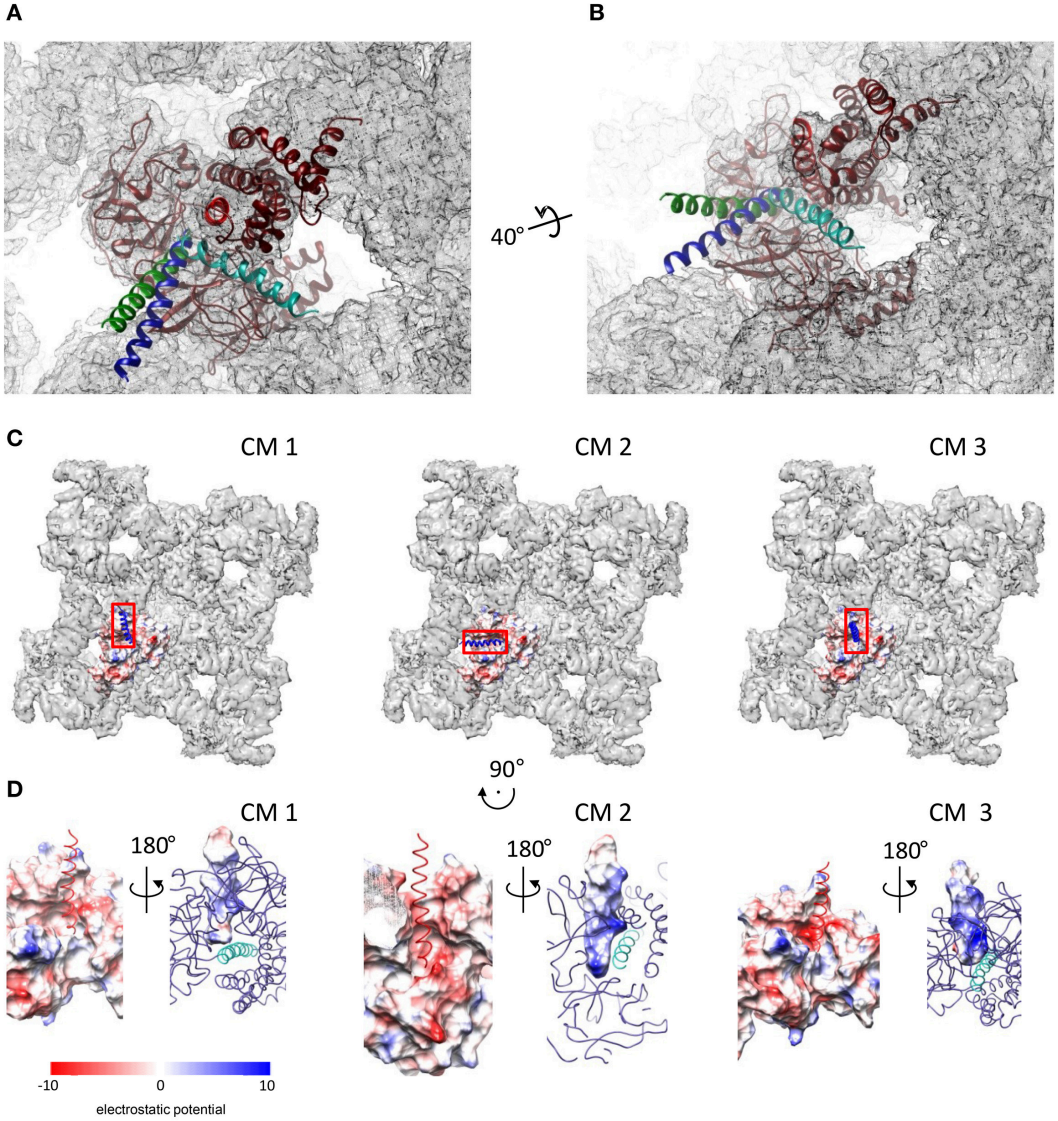
Complexes of DP_cpvtN2_ with the NTR. **(A,B)** Two views on the plausible positions of DP_cpvtN2_ in the complex with NTR. **(A)** A view along the central helix. **(B)** Oblique view. The models are positioned in the cryo-EM map of oRyR1 (EMD 2807; Yan et al., 2015). The axis and angle of rotation of the second view are shown. Models CM 1–CM 3 are shown in blue, cyan and green, respectively. **(C)** Top views of the ryanodine receptor (EMD 2807) with the docked models of NTR in the open state, shown as electrostatic potential distribution (blue, positive; red, negative) of its surface, and with bound DP_cpvtN2_ shown as a ribbon. Left to right: CM 1, CM 2, and CM 3. The rectangles correspond to the areas shown in **(D)**. **(D)** Electrostatic potential distribution of the interacting interfaces of NTR and DP_cpvtN2_. Rotation relative to panels in **(C)** is shown at the top. Left to right: CM 1, CM 2, and CM 3. In each panel, one side of the interface is shown as the electrostatic potential distribution of its surface (left—NTR; right—DP_cpvtN2_), while the complementary molecule is shown as a ribbon (left—DP_cpvtN2_; red; right—NTR, blue, with the central helix shown in a lighter shade). The complementary views (left vs. right) were created by 180° rotation around the vertical axis.

The *GRAMM-X* server provided a total of 29 conformational solutions for the DP_cpvtN2_—NTR complex. The models were checked for compatibility with the structure of the whole RyR by alignment with NTR fitted into a cryo-EM map of RyR1 (EMD 2807; Yan et al., 2015). In three conformational models (CM1–CM3), bound DP_cpvtN2_ did not sterically interfere with the RyR1 electron density. The conformations CM1–CM3 were further energy-minimized. The positions of DP_cpvtN2_ in the grooves of NTR in the closed conformation, predicted by CM1–CM3, are shown in Figures 3A,B. In all three cases DP_cpvtN2_ binds to the N-terminal domain at the cytosolic, top face of the RyR (Figure 3C).

Figure 3D shows the distribution of electrostatic potential on the complementary surfaces of NTR and DP_cpvtN2_ in the three models. The surface of DP_cpvtN2_ is positively charged due to the presence of four arginine residues oriented toward the NTR surface. The surface of the NTR groove that cradles bound DP_cpvtN2_ is charged negatively due to the presence of one serine and two glutamate residues (E50, S200, E320) in CM1, two aspartate, two serine and six glutamate residues (E40, N44, E50, N57, S136, S200, E320, E450, E521, E524) in CM2, and one aspartate and two glutamate residues (E50, N293, E320) in CM3. It is therefore apparent that the interaction between the peptide and NTR has electrostatic character.

The energies of NTR, DP_cpvtN2_ and of the DP_cpvtN2_—NTR complexes are compared in Table 2. It has to be noted that several interactions contributing to the overall energy of the complex between RyR2 and DP_cpvtN2_ are not accounted for in CM1–CM3, namely, the energy of interaction between the four NTR monomers and the energy of interaction between the N-terminal region and the remainder of the RyR2 molecule. Nevertheless, the energetic profiles from Table 2 suggest that the transition from the closed to the open conformation of the isolated NTR is energetically highly unfavorable (ΔΔ${\text{G}}_{\text{opening}}^{\text{free}}=$ +1580 kJ/mol), which is consistent with the very low open probability of the whole RyR2 in the absence of channel activators. The energies of the complex in the closed as well as in the open conformations are the smallest in CM2. In CM1 and CM3, formation of the complex in the closed conformation of NTR is energetically unfavorable, while formation of the complex in the open conformation of NTR and opening of the DP_cpvtN2_ -bound NTR are energetically favorable, suggesting low binding affinity of DP_cpvtN2_ to the closed conformation, high binding affinity of DP_cpvtN2_ to the open conformation, and high open probability of the complex. In contrast, in CM2 the energy of complex formation is highly favorable for both, open and closed conformations of NTR, which suggests high binding affinity of DP_cpvtN2_. The opening of DP_cpvtN2_ -bound NTR is energetically unfavorable, which suggests low open probability of the complex. This analysis confirms that complex formation between DP_cpvtN2_ and NTR is energetically plausible and that models CM1 and CM3 on one hand and model CM2 on the other hand predict different thermodynamic properties of DP_cpvtN2_—RyR2 binding.

**Table 2:** Energies of free and complexed NTR in the closed and open state.

	**Energy (kJ/mol)**				
**Molecule**	**Free NTR**	**DP_cpvtN2_**	**CM1**	**CM2**	**CM3**
Closed channel	−51180	−4000	−54860	−57710	−54790
Open channel	−49600	−4000	−55450	−56850	−56490
ΔΔG_opening_	+1,580	N/A	−590	+860	−1690
ΔΔ${\text{G}}_{\text{binding}}^{\text{C}}$	N/A	N/A	+320	−2530	+380
ΔΔ${\text{G}}_{\text{binding}}^{\text{O}}$	N/A	N/A	−1850	−3250	−2890

*ΔΔG, Free energy change; N/A, not applicable*.

### Compatibility between electrophysiological data and structural models

The energy differences between the open and closed conformations of CM1 and CM3 (Table 2) were consistent with high open probability of the complex (ΔΔG_opening_ < 0), in accordance with GS1. In contrast, the energy difference between the open and closed conformations of CM2 was consistent with low open probability of the complex (ΔΔG_opening_ > 0), in accordance with GS3.

The energy differences between NTR in the closed conformation with unbound DP_cpvtN2_ on one hand and the closed conformations of CM1 and CM3 on the other hand suggest a low binding affinity between DP_cpvtN2_ and NTR (ΔΔ${\text{G}}_{\text{binding}}^{\text{C}}>$ 0) in accordance with GS1; the energy difference between NTR in the closed conformation with unbound DP_cpvtN2_ and the closed conformation of CM2 suggested a high binding affinity (ΔΔ${\text{G}}_{\text{binding}}^{\text{C}}$ < 0) in accordance with GS3.

The energy differences between NTR in the open conformation with unbound DP_cpvtN2_ on one hand and the open conformation of all three models on the other hand suggested a high binding affinity between DP_cpvtN2_ and NTR (ΔΔ${\text{G}}_{\text{binding}}^{\text{O}}$ < 0). Since GS1 assumes the same binding affinity for the complex in the closed as well as in the open conformation, CM1 and CM3 are at odds with the results of bilayer experiments. The larger binding energy of CM2 in the open than in the closed conformation (ΔΔ${\text{G}}_{\text{binding}}^{\text{O}}$ − ΔΔ${\text{G}}_{\text{binding}}^{\text{C}}$ = −720 kJ/mol) is in agreement with the prediction of GS3, which postulates that the change of opening energy induced by ligand binding is reflected in the increase in binding affinity. In other words, only CM2 and GS3 are mechanistically compatible.

### Inter-domain and peptide—NTR interactions in the CM2 model

**Figure 4:**
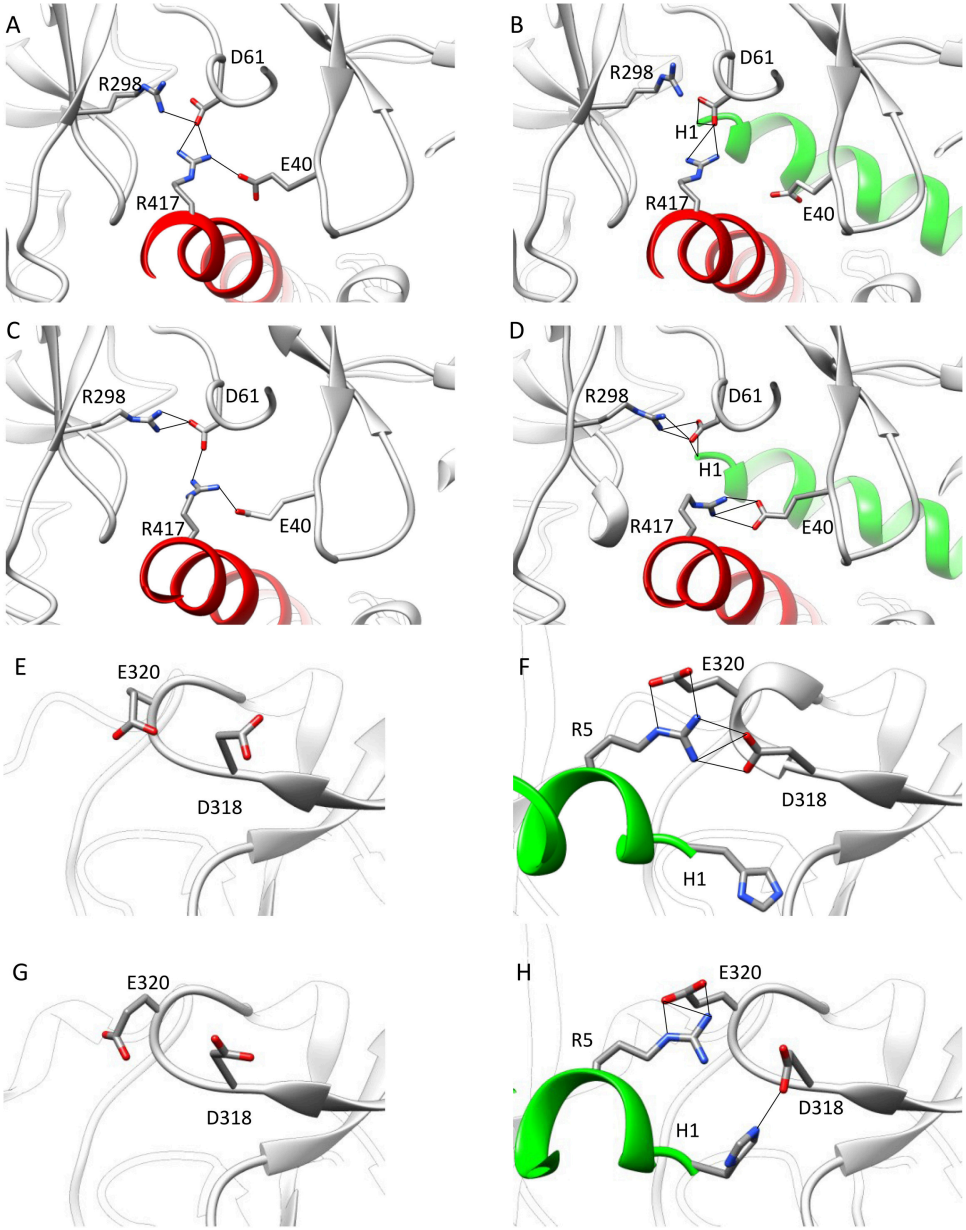
Interaction networks in NTR and CM2. **(A–D)** the NTR hydrogen bond network E40-R417-D61-R298. **(A,B)** Closed state of NTR. The interactions between E40 and R417 **(A)** are disrupted by introduction of DP_cpvtN2_ **(B)**. **(C,D)** Open state of NTR. Interaction between R417 and D61 **(C)** are disrupted but interaction between D61 and R298 is strengthened by introduction of DP_cpvtN2_ **(D)**. **(E–H)** the CM2 hydrogen bonding network. **(E,F)** closed state of NTR; **(G,H)** open state of NTR. A hydrogen bonding network is formed in CM2 **(F,H)** between D318 and E320 from domain B and H1 and/or R5 from DP_cpvtN2_. The central helix is shown in red; DP_cpvtN2_ is shown in green.

To find out whether CM2 is compatible with the “domain switch” mechanism involving domains A and B of the N-terminal region, the inter-domain and peptide-NTR interactions were analyzed further. The interactions between amino acids R417, R298, E40, and D61 were previously found to be important for the stability of NTR (Borko et al., 2014), and are summarized for individual NTR molecules in Table 3. In the closed conformations (Figures 4A,B), interactions present in NTR—the hydrogen bond between amine group of R417 and carboxyl oxygen of E40, and the hydrogen bond between the amine group of R298 and carboxyl oxygen of D61 were disrupted in CM2 due to conformational changes evoked by formation of hydrogen bonds between the main-chain nitrogen of H1 in DP_cpvtN2_ and the carboxyl oxygens of D61 in NTR. In the open conformation (Figures 4C,D), the overall number of hydrogen bonds between R417, R298, E40, and D61 was higher for CM2 than for the free NTR: the amine nitrogens of R298 and the carboxyl oxygens of D61 formed two hydrogen bonds in NTR but three hydrogen bonds in CM2, the amine nitrogens of R417 and the carboxyl oxygens of E40 formed one hydrogen bond in NTR but three hydrogen bonds in CM2, and one hydrogen bond was present between the amine nitrogens of R417 and the carboxyl oxygens of E61 in NTR but not in CM2. As in the closed conformation, these changes in hydrogen bonding were accompanied by formation of hydrogen bonds between the main-chain nitrogen of H1 in DP_cpvtN2_ and the carboxyl oxygens of D61 in NTR. A major conformational change was also observed in the region encompassing D318 and E320, in which no important inter-domain interactions were present in NTR (Figures 4E,G) but in CM2 a network of H-bonds (Figures 4F,H) was formed between the NTR carboxyl oxygens of D318 and E320 the DP_cpvtN2_ amine and imine nitrogens of R5 (5 hydrogen bonds in the closed conformation and 3 hydrogen bonds in the open conformation) and the DP_cpvtN2_ imine nitrogen of H1 (1 hydrogen bond in the open conformation).

**Table 3:** Hydrogen bonding network R298-E40-R417-D61 of the wild type and mutant NTR in the closed and open state.

	**Energy (kJ/mol)**																	
**Molecule**	**WT**		**CM2**		**R414C**		**R414L**		**T415R**		**I419F**		**R420W**		**R420Q**		**L433P**	
**Hydrogen bond**	**C**	**O**	**C**	**O**	**C**	**O**	**C**	**O**	**C**	**O**	**C**	**O**	**C**	**O**	**C**	**O**	**C**	**O**
R298 NH1 → D61 OD1	×			×	×		×		×		×		×		×		×	
R298 NH1 → D61 OD2		×				×		×		×		×		×		×		×
R298 NH2 → D61 OD1				×														
R298 NH2 → D61 OD2		×		×		×		×		×		×		×		×		×
R417 NH1 → D61 OD1	×	×	×		×	×	×	×	×		×		×	×	×	×	×	
R417 NH1 → E40 OE1				×														
R417 NH1 → E40 OE2						×		×		×		×		×		×		×
R417 NH2 → E40 OE1				×														
R417 NH2 → E40 OE2	×	×		×	×		×		×		×		×		×		×	
R417 NH2 → D61 OD1	×		×		×	×	×	×	×		×	×	×	×	×	×	×	×

*Hydrogen bonds that fulfill the criteria (Mills and Dean, 1996) with constraints relaxed by 0.4 Å and 30° are shown by “ ×.” C, closed state; O, open state. Arrows point from donor to acceptor*.

The peptide formed a number of further hydrogen bonds and ionic and hydrophobic contacts with amino acid residues from domains A and B (Table 4). Out of 11 H-bonds in the closed conformation, 4 remained present in the open conformation while 5 new H-bonds were formed. The hydrogen bonds between the peptide and the domains A, B of NTR (Table 4) did not have a counterpart in equivalent H-bonds between the central helix and the domains A, B of free NTR (column 1 and 3 of Table 5). As an example, the amine nitrogen of R414 formed a hydrogen bond with the main chain oxygen of S126 from the A domain of NTR in the free NTR as well as in CM2, while the amine nitrogens of the corresponding peptide amino acid R5 formed hydrogen bonds with carboxyl oxygens of E320 and D318 from the B domain of NTR. The number of H-bonds between the peptide and domains A, B was comparable to the number of H-bonds between the central helix and domains A, B, which decreased from 12 to 9 upon peptide binding in the closed conformation (columns 1 and 2 of Table 5) and remained at 7 in the free NTR as well as in CM2 in the open conformation (columns 3 and 4 of Table 5).

**Table 4:** Hydrogen bonding between DP_cpvtN2_ and domains A, B of NTR.

**Closed state**	**Open state**
H1(410) N → D61 OD1	H1(410) N → D61 OD1
H1(410) N → D61 OD2	H1(410) N → D61 OD2
H1(410) N → K 304 O	
	H1(410) NE2 → D318 OD1
H1(410) O ← K 319 NZ	
	E2(411) OE1 ← K319 N
R5(414) NE → E320 OE1	R5(414) NE → E320 OE1
R5(414) NH1 → D318 OD1	
R5(414) NH1 → D318 OD2	
R5(414) NH2 → D318 OD2	
	R5(414) NH2 → E320 OE1
R5(414) NH2 → E320 OE2	R5(414) NH2 → E320 OE2
	V14(423) O → R137 NH1
	V14(423) O → R137 NH2
F17(426) ← R137 NE	
F17(426) ← R137 NH2	

*For DP_cpvtN2_ residues, the numbers in parentheses correspond to the sequence numbers of the respective residue in the central helix. The arrows point from the donor to the acceptor*.

**Table 5:** Hydrogen bonding between the central helix and domains A, B of NTR.

**Closed state**		**Open state**	
**NTR**	**CM2**	**NTR**	**CM2**
		H410 NE2 → S124 O	H410 NE2 → S124 O
E412 OE1 → R235 NH1	E412 OE1 ← R235 NH1		
E412 OE2 → R235 NH1	E412 OE2 ← R235 NH1		
		S413 OG → R276 NE	
		S413 OG → R276 NH2	
R414 NH1 → S126 O	R414 NH1 → S126 O	R414 NH1 → S126 O	R414 NH1 → S126 O
A416 O → T301 OG1			
R417 NE → Y125 OH	R417 NE → Y125 OH		
R417 NH1 → D61 OD1	R417 NH1 → D61 OD1	R417 NH1 → D61 OD1	
	R417 NH1 → D61 OD2		
			R417 NH1 → E40 OE1
			R417 NH2 → E40 OE1
R417 NH2 → E40 OE2		R417 NH2 → E40 OE2	R417 NH2 → E40 OE2
R417 NH2 → D61 OD1			
R417 NH2 → Y125 OH	R417 NH2 → Y125 OH		
R420 NE → T301 O			
R420 NH2 → T301 O			
S421 OG → E40 OE1	S421 OG → E40 OE2	S421 OG → E40 OE1	S421 OG → E40 OE1
			S421 OG → E40 OE2
	S421 OG → Y123 OH		

*The arrows point from the donor to the acceptor*.

Hydrophobic and ionic interactions of domains A, B with the domain peptide and with the central helix were also different: In the CM2 complex, DP_cpvtN2_ amino acids I10 and V14 formed hydrophobic contacts with the respective amino acids L46 and F48 from the A domain of the NTR, and DP_cpvtN2_ amino acid R5 (corresponding to R414 of the central helix) formed ionic contacts with D318 and E320 from the B domain of the NTR. On the other hand, no hydrophobic contacts were present between the central helix and domains A, B, since the hydrophobic side of the central helix was positioned toward domain C; ionic contacts were formed between the central helix amino acid R417 and E40 and D61 from the A domain and between the central helix amino acid E412 and R235 from the B domain. Thus, the nature of hydrogen bonds, ionic and hydrophobic contacts between the central helix or the domain peptide on one hand and domains A, B on the other has no similarities. The similarity between the effect of DP_cpvtN2_ and that of central helix mutations on RyR activity is therefore not caused by competition between the domain peptide and the corresponding central helix amino acids for the binding site at the “domain zipper,” as postulated for the “domain switch” mechanism (Ikemoto and Yamamoto, 2000; Yamamoto et al., 2000; Tateishi et al., 2009; Suetomi et al., 2011). It is conceivable, that RyR2 activation by DP_cpvtN2_ and its destabilization by central helix mutations arises from similarities between the energy changes caused by complex formation and those caused by mutation of amino acids in the central helix.

### Models of central helix mutants of NTR

**Figure 5:**
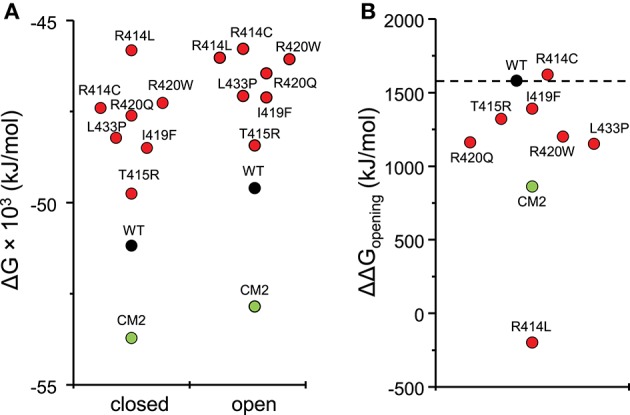
Energies of wild type and mutant NTRs in the closed and open state. **(A)** Energies of individual molecules in the closed and open state; **(B)** opening energies of individual molecules.

To compare the energy changes caused by complex formation and by mutation of amino acids in the central helix, we have created models of NTR, each with one of the seven reported mutations, in the closed and open conformation and optimized them by energy minimization. Total energies of individual molecules of WT and mutant NTRs in the closed and open conformation are compared in Figure 5 (the numerical values are also given in Table 6). Complex formation between NTR and DP_cpvtN2_ (Table 2) is energetically favorable since the energy of both, the closed and the open conformation is more negative than the energy of the free NTR and free DP_cpvtN2_. This is reflected in the high binding affinity of DP_cpvtN2_ to the NTR. On the contrary, all models of mutant NTRs show higher, less favorable energies than the wild type NTR (Figure 5A). Thus, the energy changes induced by complex formation and by amino acid mutations are in the opposite direction.

**Table 6:** Energies of wild type and mutant NTR in the closed and open state.

	**Energy (kJ/mol)**								
**Molecule**	**WT**	**CM2**	**R414C**	**R414L**	**T415R**	**I419F**	**R420W**	**R420Q**	**L433P**
Closed channel	−51,180	−57,710	−47,400	−45,820	−49,750	−48,500	−47,260	−47,610	−48,220
Open channel	−49,600	−56,850	−45,780	−46,020	−48,430	−47,110	−46,060	−46,450	−47,070
ΔΔG_opening_	+1,580	+860	+1,620	−200	+1,320	+1,390	+1,200	+1,160	+1,150

*ΔΔG, Free energy change*.

**Figure 6:**
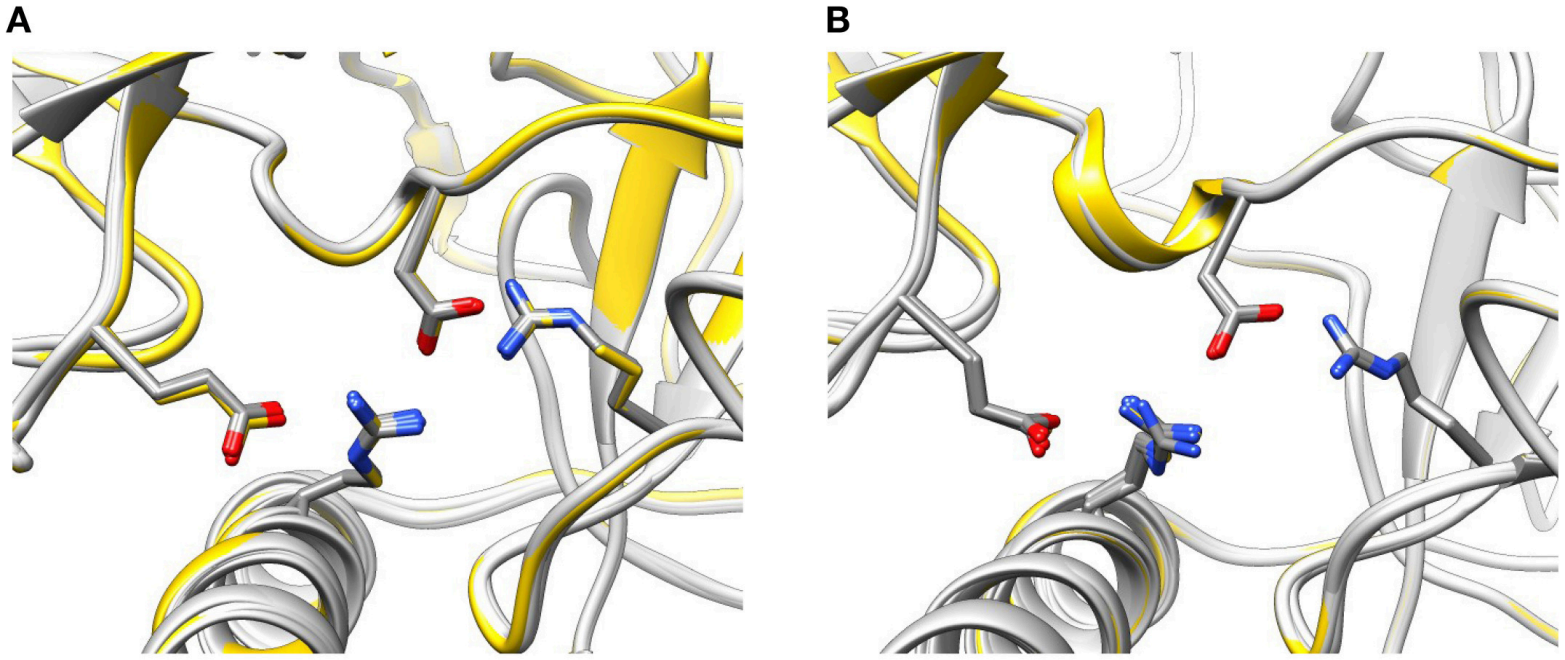
Configuration of the interaction network D61—R417—E40—R298 in mutant NTRs. **(A)** Wild type (yellow) and mutant (light gray) NTR models in the closed **(A)** and open state **(B)**.

The conformation of the amino acids E40, D61, R298, and R417 involved in formation of the hydrogen-bonding network showed no significant differences from the wild-type NTR in the models of mutant NTRs either in the closed or in the open conformation (Figure 6). In the closed conformation, the number of hydrogen bonds remained unchanged (n_HB_ = 4) in all mutants. In the open conformation, the number of hydrogen bonds remained unchanged (n_HB_ = 4) in three mutants (I419F, L433P, and R414L), it increased by one in two mutants (R414C and R420W) and it decreased by one in one mutant (T415R) (Table 3). The configuration of the hydrogen-bonding network involving the central helix cannot therefore be the main reason for the increased total energy of mutant NTRs.

To determine, which interactions are most important for the energy changes observed upon the closed/open conformational transition and upon mutation, we have tested linear models incorporating the number of intramolecular interactions (including hydrogen bonds) between and within subdomains (i.e., domains A, B, C and the central helix). The complex of NTR with DP_cpvtN2_ was not included into the analysis, since its total energy is heavily affected by intermolecular interactions that are not present in the free NTR molecules. The *energy difference* between the open and closed conformation of WT and mutant NTRs could not be directly expressed as a function of the number of intramolecular interactions (in all tested models, the prediction for at least two out of 8 molecules had a relative error of >20%). The *total energy* of NTR and its mutants in both the closed and the open conformation could be satisfactorily approximated (relative error of <5%; *R* = 0.62, CV = −0.022), assuming a linear dependence on the number of ionic interactions between subdomains (${\text{N}}_{\text{I}}^{\text{E}}$, F = 12.3, *P* = 0.004) and on the number of hydrogen bonds between amino acid side chains (${\text{N}}_{\text{HB}}^{\text{SC}}$; *F* = 9.3, *P* = 0.009):

(4) $$\Delta \text{G}=-26039-238{\text{N}}_{\text{I}}^{\text{E}}-119{\text{N}}_{\text{HB}}^{\text{SC}}.$$

That is, the increased energies of mutant NTRs were mostly due to a decreased number of ionic interactions between the subdomains and a decreased number of hydrogen bonds between amino acid sidechains. Hydrogen bonds involving main-chain atoms, ionic interactions within subdomains, and hydrophobic, aromatic and cation-π interactions contributed to the total energy of the molecules on average by −26039 kJ/mol. Contributions of these interactions to the total energy could not be further dissected, most probably because of their low number (aromatic and cation-π interactions) or because of a smaller variation between individual studied molecules (hydrogen bonds involving main chain atoms, ionic interactions within subdomains, and hydrophobic interactions).

In the closed conformation, mutations did not change the number of inter-domain ionic contacts (${\text{N}}_{\text{I}}^{\text{E}}$ = 15 for WT and ${\text{N}}_{\text{I}}^{\text{E}}$ = 13.9 ± 0.7, N = 7 for mutant NTRs) but decreased the number of hydrogen bonds between amino acid side-chains (${\text{N}}_{\text{HB}}^{\text{SC}}$ = 178 for WT and 157 ± 2.7, N = 7 for mutant NTRs, *P* < 0.001), which is in accordance with the higher total energy of mutant NTRs when compared with the wild type. In the open conformation, the number of inter-domain contacts was not significantly changed by mutations (${\text{N}}_{\text{I}}^{\text{E}}$ = 12 for WT and 11.7 ± 0.2 for mutant NTRs) and the number of hydrogen bonds between amino acid side-chains in mutant NTRs was smaller only slightly albeit significantly (${\text{N}}_{\text{HB}}^{\text{SC}}$ = 154 for WT and 151 ± 0.7, *N* = 7 for mutant NTRs, *P* < 0.01).

While mutant NTRs differed from CM2 in having a higher total energy than the wild-type NTR, the energy differences between the open and closed conformation of the NTR showed a similar tendency for both, CM2 and mutant NTRs (Figure 5B). With the exception of R414C, the mutant NTRs shared with the complex a lower energy necessary for channel opening (Figure 5B). The value of ΔΔG_opening_ was 1,091 ± 224 kJ/mol (mean ± *SE*) for mutant NTRs on average and 860 kJ/mol for CM2, compared with 1,580 kJ/mol for wild-type NTR. These data are consistent with a higher open probability for channels with mutant NTRs as well as for channels with bound DP_cpvtN2_.

## Discussion

This study examined the effect of the domain peptide DP_cpvtN2_ with a sequence identical to the central helix of the RyR2 NTR on the activity of ryanodine receptors using electrophysiology (rat RyR2) and structural modeling (human RyR2). The sequence of the central helix is fully conserved in many mammalian species including rat and human, and the sequence identity between rat and human RyR2 NTR is 96.5%; this allowed us to use rat RyR2 for the experiments and human RyR2 NTR for structural modeling. Electrophysiological experiments demonstrated activation of rat cardiac ryanodine receptors by DP_cpvtN2_. Examination of structural models of human NTR-DP_cpvtN2_ complexes in the closed and open conformation has shown that DP_cpvtN2_ may bind to the RyR2 NTR by an allosteric mechanism that increases the probability of the open conformation. However, the interactions between DP_cpvtN2_ and NTR predicted by structural modeling are at odds with the classical “domain zipper” mechanism, i.e., DP_cpvtN2_ and the central helix did not compete for a common binding site. The similarity between the effect of the peptide and the presumed effect of mutations may arise due to a similar effect of peptide binding and amino acid mutation on the energy difference between the open and closed conformation of the NTR.

DP_cpvtN2_ activated the RyR2 channel by inducing rare but very long openings that never occurred in the absence of the peptide. The increase of RyR open probability with increasing concentration of DP_cpvtN2_ suggests that there is a dynamic equilibrium between the peptide-free and peptide-bound ryanodine receptor, that is, that the interaction between the channel and the peptide is readily reversible; otherwise, the effect of the peptide should be all-or-none, as observed previously for the effect of DIDS on RyR2 activity (Zahradnikova and Zahradnik, 1993). In this respect, the effect of DP_cpvtN2_ was similar to that of the central domain peptide DP_cpvtC_ (Faltinova and Zahradnikova, 2013).

In all spatially plausible models of peptide binding to the N-terminal region, DP_cpvtN2_ electrostatically interacted with domains A and B of the NTR by its N-terminal end. However, only the CM2 model and the gating scheme GS3 were fully compatible with both the electrophysiological and structural modeling data and suggested an allosteric gating mechanism of peptide-NTR interaction. This is in line with the observations of the effect of other RyR ligands, namely, cytosolic (Zahradnik et al., 2005) and luminal Ca^2+^ (Tencerova et al., 2012), ATP (Tencerova et al., 2012), and of the peptide DP_cpvtC_ from the central domain (Faltinova and Zahradnikova, 2013) that were shown to activate the ryanodine receptor by an allosteric mechanism.

In our simulations, the energy differences between free and complexed NTR and between the open and closed conformations were caused by subtle changes in the network of hydrogen bonds comprising amino acids E40 and D61 from domain A, R298 from domain B, and R417 from the central helix of the NTR, which were previously shown to be essential for the stability of the NTR (Kimlicka et al., 2013b; Borko et al., 2014). Additionally, a new network of hydrogen bonds was formed between the carboxyl oxygens of D318 and E320 of domain B and the amine/imine nitrogens of R5 and/or H1 of DP_cpvtN2_ with more hydrogen bonds in the closed than in the open conformation.

The effect of central helix mutations that cause clinically important arrhythmias on RyR2 open probability has not been previously examined in detail. The available data suggest an effect of mutations R420Q, R420W, and L433P on cellular calcium handling (Thomas et al., 2004; Tang et al., 2012; Okudaira et al., 2014; Domingo et al., 2015; Novak et al., 2015). It is generally accepted that most arrhythmogenic RyR2 mutations are gain-of-function, i.e., they increase RyR2 activity (Lehnart et al., 2004; Meli et al., 2011), the probability of occurrence of calcium waves (Fernandez-Velasco et al., 2009) or facilitate store-overload induced calcium release (Jiang et al., 2005; Jones et al., 2008). Previously, the gain of function induced by RyR2 mutations has been explained by the “domain switch” mechanism (Ikemoto and Yamamoto, 2000; Yamamoto et al., 2000; Tateishi et al., 2009; Suetomi et al., 2011). Our modeling studies show that the effect of DP_cpvtN2_ on RyR activity cannot be caused by this mechanism, since competition between DP_cpvtN2_ and the central helix amino acids for a common binding site could be excluded. Thus, why both the DP_cpvtN2_ (this work) and the mutations in the central helix (Thomas et al., 2004; Tang et al., 2012; Okudaira et al., 2014; Domingo et al., 2015; Novak et al., 2015) induce gain-of-function behavior of RyR2? Examination of the models of individual central helix mutants in the closed and open conformation revealed that their total energy is higher (less negative) than that of wild-type NTR. This is not surprising, since several NTRs of RyR1 and RyR2 with an incorporated mutation showed a lower thermodynamic stability than the corresponding wild type NTR. For instance, mutations C36R, R45C, R402G, L14R, G216E, I404M, and V219I of the rabbit RyR1 NTR (Kimlicka et al., 2013a) and mutation I419F of the human RyR2 NTR (Borko et al., 2014) had a significantly lower melting temperature than the corresponding wild type NTR. On the other hand, melting temperature of NTRs with RyR1 mutations D61N and G249R or RyR2 mutation R420W was not significantly different from that of the corresponding wild type NTR (Kimlicka et al., 2013a; Borko et al., 2014). In contrast, the decrease of total energy upon binding of DP_cpvtN2_ to NTR in CM2 is consistent with the observed high affinity of DP_cpvtN2_ toward RyR2 as well as with the low EC50 of other domain peptides affecting RyR activity [20–60 μM for peptides DP1 and DP4 (Ikemoto and Yamamoto, 2000); ~10 μM for DPc10 (Laver et al., 2008); 20 μM for DP_cpvtC_ (Faltinova and Zahradnikova, 2013)], which points to high thermodynamic stability of peptide complexes with RyR. Thus, the total energies of the studied structural models do not explain the similarity between the effect of mutations and the effect of the peptide DP_cpvtN2_ on the activity of RyR2.

A common feature of the model of the DP_cpvtN2_-NTR complex (CM2) and models of central helix mutants of NTR was their increased thermodynamic stability in the open conformation relative to the closed conformation (with the exception of the mutant R414C; Figure 5B). In the DP_cpvtN2_-NTR complex, this could be partially explained by the lower stability of the inter-domain hydrogen-bonding network E40-R417-D61-R298 in the closed but not in the open conformation. The energy changes in mutant NTRs could be attributed to a lower number of hydrogen bonds between amino acid side chains, relative to that in the wild type NTR, in the closed but not in the open conformation.

It has been proposed that the ryanodine receptor central rim (Lanner et al., 2010), composed of a tetramer of domains A and B of the NTR (Yuchi and Van Petegem, 2011; Borko et al., 2014), plays an important role as a suppressor of channel opening (Kimlicka et al., 2013a; Yuchi and Van Petegem, 2016). In the closed conformation, the central rim is kept in a tight conformation by interactions between the A and B domains of the same monomer (Yuchi and Van Petegem, 2011; Borko et al., 2014) as well as between domain A of one monomer and domain B of the neighboring monomer (Kimlicka et al., 2013a; Borko et al., 2014; Yuchi and Van Petegem, 2016). In the open conformation, the NTRs assume a more relaxed conformation tilted up and outwards (Kimlicka et al., 2013a; Borko et al., 2014; Yuchi and Van Petegem, 2016). Since RyR gating is under allosteric control, modifications that stabilize the relaxed conformation facilitate channel opening. It has been speculated that mutations in the RyR “hot spot loop” (Amador et al., 2009) that lie at the inter-monomer interface of domain A may be responsible for the facilitated opening of the respective mutant RyR channels (Kimlicka et al., 2013a; Yuchi and Van Petegem, 2016). Here we show that increased RyR open probability can be achieved also by conformational changes occurring within the N-terminal region. Importantly, our modeling studies show that the stability of the N-terminal region, as defined by its free energy, may not be important for the gating behavior of the RyR: in our models, the free energies of the NTR in both the closed and the open conformation increased in the order CM2 < NTR^WT^ < NTR^T415R^ < NTR^R414L^, but the free energy of opening increased in the order NTR^R414L^ < CM2 < NTR^T415R^ < NTR^WT^. Facilitation of RyR opening in the case of the DP_cpvtN2_-NTR complex (CM2) was caused by selective destabilization of inter-domain interactions in the closed conformation, as previously suggested for the central rim region (Yuchi and Van Petegem, 2011, 2016; Kimlicka et al., 2013a; Borko et al., 2014) as well as for the “domain switch” region (Ikemoto and Yamamoto, 2000, 2002; Yamamoto et al., 2000). In contrast, in models of NTR central helix mutants, destabilization of the closed conformation occurred by a decreased number of hydrogen bonds between amino acid side-chains both within and between domains. In the open conformation, the changes in the number of hydrogen bonds between amino acid side-chains induced by peptide binding or amino acid mutation bonding were much less prominent.

It has to be noted that the presented simulations, while showing plausibility of DP_cpvtN2_ binding to the NTD, do not rule out other sites of DP_cpvtN2_ interaction with the cytosolic part of the RyR2 molecule. Rather, they provide a strong indication that the deleterious effect of the central helix mutations on RyR2 function is not due to a defect in inter-domain interactions but rather in their effect on the energetics of the closed-open transition of the NTD that might facilitate the closed-open transition of the whole channel. To resolve this question, further studies are necessary, such as direct measurements and/or molecular dynamics simulations of the thermodynamics of interactions between the involved domains and domain peptides.

## Author contributions

AZ conceived the study, AF performed the bilayer experiments, NT and MA performed CD spectroscopy experiments, AF, NT, MA, and AZ analyzed the data, AZ performed bioinformatics analyses and molecular modeling, AZ and JS evaluated the molecular models, AZ and JS wrote the first draft of the manuscript, and all authors contributed to the final wording of the manuscript.

### Conflict of interest statement

The authors declare that the research was conducted in the absence of any commercial or financial relationships that could be construed as a potential conflict of interest.
